# Polish validation of soft skills questionnaire for nurses

**DOI:** 10.3389/fpubh.2025.1597455

**Published:** 2025-07-29

**Authors:** Katarzyna Tomaszewska, Krystyna Kowalczuk, Marek Sobolewski, Bożena Majchrowicz

**Affiliations:** ^1^Department of Nursing, Institute of Health Protection, The Bronisław Markiewicz Academy of Applied Sciences, Jarosław, Poland; ^2^Department of Integrated Medical Care, Medical University of Białystok, Białystok, Poland; ^3^Faculty of Management, Rzeszow University of Technology, Rzeszow, Poland; ^4^Department of Nursing, Institute of Health Protection, State Academy of Applied Sciences, Przemyśl, Poland

**Keywords:** nurse, soft skills, questionnaire, validation, nursing

## Abstract

**Introduction:**

The professional competence of nurses is a pivotal factor in ensuring patient safety and significantly influences the quality of care delivered. In line with the guidelines of the International Council of Nurses and the World Health Organization, educational programs for nursing professionals must emphasize the development of both psychosocial and psychomotor competencies to maintain high training standards. Additionally, effective quality management in healthcare institutions necessitates continuous monitoring of nurses' competencies, encompassing not only practical skills but also interpersonal abilities.

**Aim:**

The aim of this study was to translate and adapt the Soft Skills Questionnaire for Nurses into a Polish-language version and to evaluate its reliability and validity as an instrument for assessing nurses' soft skills.

**Material and methods:**

The validation study was conducted from April to June 2024 among a cohort of 496 actively practicing nurses in Poland. A two-stage validation process was employed. The first stage involved the linguistic and cultural adaptation of the original questionnaire into Polish, following formal authorization from the original authors. The second stage entailed a psychometric evaluation of the translated instrument. Participants completed the questionnaire twice, with a 1-month interval between administrations to assess test-retest reliability. The internal consistency of each subscale was evaluated using Cronbach's alpha coefficient. Agreement between the two sets of responses was analyzed by calculating descriptive statistics for both time points, followed by assessment of the differences using the Wilcoxon signed-rank test. The strength and direction of association between the two administrations were examined using Spearman's rank correlation coefficient. Additionally, test-retest reliability was assessed using the intraclass correlation coefficient (ICC), providing further evidence of the tool's stability over time.

**Results and conclusions:**

The questionnaire comprised three distinct subscales designed to evaluate key domains of soft skills: communication skills (NCS—Nursing Communication Skills), confidentiality (CON—Confidentiality), and management and emotional intelligence (MEI—Management and Emotional Intelligence). The internal consistency of the NCS subscale was relatively high, with a Cronbach's alpha coefficient of 0.669, approaching the commonly accepted threshold of 0.70 for satisfactory reliability. The CON subscale demonstrated a slightly lower internal consistency (α = 0.601), while the MEI subscale yielded the lowest internal consistency (α = 0.544), suggesting potential variability in item coherence within this domain. Despite these moderate reliability indices, all additional statistical analyses supported the robustness of the questionnaire. The Wilcoxon signed-rank test revealed no statistically significant differences between the test and retest administrations (*p* > 0.05), indicating the absence of systematic response shifts over time. Furthermore, Spearman's rank correlation coefficients between the two administrations were exceptionally high (r_s_ > 0.90), and test-retest reliability, as assessed by the intraclass correlation coefficient (ICC), also exceeded 0.90 across all subscales. These findings indicate that the Polish adaptation of the *Soft Skills Questionnaire for Nurses* demonstrates strong temporal stability and satisfactory psychometric performance, supporting its suitability for use in both clinical assessments and empirical research on soft skills within the nursing profession.

## 1 Introduction

Nursing personnel are a vital professional group within the healthcare system. Through their continuous provision of direct and indirect patient care, nurses significantly influence patient outcomes and the overall quality of care. Despite their crucial role, a global shortage of nursing staff persists, with the average age of nurses, for example, in Poland, being approximately 54 years.

Possessing the necessary competencies is fundamental to delivering high-quality services in nursing. The profession demands a unique set of skills due to its inherent responsibility and the constant interpersonal interactions involved. In the context of patient care, both social and professional competencies play a pivotal role (1–3). These competencies are particularly critical in light of the increasing complexity of healthcare services. Simultaneously, advances in medical technology and rising patient expectations for quality care necessitate that nurses acquire and refine a broad range of soft skills—also referred to as human or social-emotional skills (4, 5). These skills complement professional competencies and are becoming increasingly important in daily clinical practice.

Soft skills enhance professionalism and support the development of critical abilities such as communication, teamwork, problem-solving, and critical thinking (6). These competencies are often cultivated through experience and learning, with the International Council of Nurses defining them as skills enabling decision-making in critical situations, ensuring patient safety, and mastering essential clinical tasks (7).

The Nurse Journal identifies ten key soft competencies essential for nursing practice, including communication, critical thinking, compassion, responsibility, stress management, physical fitness, professionalism, and teamwork (8). These skills not only support collaboration with other healthcare professionals but also foster effective teamwork and the ability to give and receive constructive feedback, thereby enhancing the quality of care provided (9, 10).

Building on this perspective, Nilsson et al. emphasize that soft competencies contribute to a nurse's ability to adapt professionally. Performing tasks under stress can lead to better patient care outcomes, increased job satisfaction, and reduced burnout and absenteeism. The European Strategy for Nursing and Midwifery, developed by the World Health Organization and aligned with the guidelines of the International Council of Nurses, advocates for nursing education programs that ensure the acquisition of both psychosocial and psychomotor skills necessary for professional practice (11–13).

Conversely, Heydari et al. (14) argue that the level of nurses' competencies directly impacts the quality of patient care, with lower levels potentially leading to frustration and adverse outcomes. They stress the importance of continuous monitoring of nurses' competencies, encompassing both practical and soft competencies, to ensure quality management (15).

Song et al. (16) highlight that “commitment to quality patient care refers to soft skills that include self-awareness, flexibility, communication, and critical thinking.” Sancho-Cantus et al. (17) define soft skills as a set of abilities that optimize individual performance, a perspective supported by other research (18). The American Association of Critical-Care Nurses underscores soft skills as integral to safe patient care and fostering a positive work environment, emphasizing the importance of personal traits, character, and behaviors (19).

Ernawati (20) characterize soft skills as those facilitating workplace effectiveness and meeting the demands of the nursing profession. The publication *Lost in Translation: Soft Skills Development in European Countries* categorizes soft skills into personal, social, individual, and learning skills. Soft competencies not only enhance nursing practice but also contribute to career achievement (21). In reviewing the relevant literature, the authors did not identify any standardized questionnaires for assessing nurses' soft skills. Therefore, in order to fill this knowledge gap, the authors conducted a review of published journal articles to identify existing tools in this area and to evaluate the possibility of their potential application.

The aim of this paper was to translate and adapt the Soft Skills Questionnaire for Nurses into a Polish-language version and to evaluate its reliability and validity as an instrument for assessing nurses' soft skills.

## 2 Material and methods

### 2.1 Research design

The study aimed to validate the Polish version of the Soft Skills Questionnaire for Nurses (SSQN), with a focus on assessing its theoretical relevance, reliability, and criterion validity. The research design incorporated a two-stage evaluation:

1. Translation and Verification: Translation of the questionnaire and assessment of its comprehensibility, clarity, and acceptability in Polish.
2. Psychometric Evaluation: Pilot testing for content validity and a methodological study to examine the questionnaire's psychometric properties.

### 2.2 Research tools

The Soft Skills Questionnaire for Nurses (SSQN), developed by Aridi et al. (19) was used as the primary instrument for this study. The questionnaire is composed of two sections. The first section collects sociodemographic data, providing essential background information on the participants like: gender, age, work experience, education, and knowledge of foreign languages. The second section includes 25 items presented randomly and measured on a 5-point Likert scale, where 1 indicates “strongly disagree” and 5 indicates “strongly agree”.

The items in the second section are grouped into three dimensions, each addressing a specific area of soft skills. The first dimension focuses on communication skills, assessed through 13 items. The second dimension evaluates confidentiality, covered by 6 items, and the third dimension examines management and emotional intelligence, also represented by 6 items. The original developers of the tool validated it using a sample of 56 nurses employed at two hospitals.

The SSQN (Soft Skills Questionnaire for Nurses) was selected due to its dedicated focus on the specific nature of nursing work and its inclusion of three key areas of soft skills: communication, confidentiality, and emotional intelligence. Unlike general psychological or managerial tools, the SSQN was developed with the professional practice of nurses in mind and reflects real clinical situations. An additional advantage of the SSQN is its compact format (25 items), which allows for easy application in clinical settings without placing an excessive burden on respondents. While other tools exist that assess individual components (e.g., self-assessment of emotional competence or interpersonal skills), the SSQN is one of the few instruments that integrates these dimensions into a coherent structure tailored to the context of nursing.

### 2.3 Translation process and cultural adaptation

The translation and cultural adaptation of the SSQN involved a structured, multi-phase process to ensure linguistic and cultural appropriateness. First, permission was obtained from the original authors to translate the questionnaire into Polish. Two independent translators were engaged for this task, with one translator possessing expertise in medical terminology and familiarity with the questionnaire's purpose. Next, the translated version was back-translated into English by native bilingual speakers proficient in both Polish and English. This step was undertaken to ensure equivalence across the two languages. A comparison of the original and back-translated versions followed, with discrepancies addressed to maintain the integrity of the tool.

To further ensure the accuracy and clarity of the translated version, a preliminary pilot study was conducted with five nurses who had at least 5 years of professional experience. These individuals were familiar with the issue of soft skills in clinical practice and agreed to serve as experts evaluating the comprehensibility and cultural relevance of the questionnaire items. Moreover, these nurses were asked to evaluate the instructions and individual questions for comprehensibility and alignment with workplace practices. Feedback from this stage led to minor adjustments to the wording of one question.

Subsequently, a second pilot study was conducted with 30 nurses, also with at least 5 years of experience, using the revised version of the questionnaire. This phase aimed to validate the final Polish version of the SSQN. This iterative process ensured that the tool was both linguistically and culturally appropriate for use in Poland.

A standard two-stage procedure for the adaptation and validation of the instrument was applied, in accordance with international guidelines, including the WHO recommendations (Guidelines for Translation and Adaptation of Instruments). The process involved a pilot study, forward–backward translation by two independent translators, linguistic and cultural consultations with a group of nurses and a methodology expert, as well as cultural adaptation—adjusting expressions and contextual references to reflect the realities of the Polish healthcare system.

### 2.4 Psychometric evaluation

The validation process of the questionnaire consisted of two stages. The first stage, conducted after obtaining permission from the original authors, involved translating the instrument into Polish. The second stage focused on assessing the psychometric properties of the translated tool.

The study was carried out between April and June 2024 in Poland, involving a sample of 496 actively practicing nurses. The questionnaires were appropriately coded in an anonymous manner, allowing for the pairing of test and retest results without compromising participants' privacy. The instructions provided clear guidelines on how to respond and emphasized confidentiality. The assessments were conducted in designated rooms (staff break rooms) where a quiet environment free from external disturbances and time pressure was ensured.

The authors decided to study nurses employed at two hospitals with comparable staffing levels within the nursing profession, located in the same region, and for which approval to conduct the study was obtained. Although the sample includes only two hospitals, it was assumed that their functional diversity, along with the wide range of represented positions and professional experience, would allow for the collection of data with generalizable value. A total of 600 questionnaires were administered – 300 in each hospital—to eligible nurses present during their scheduled shifts. Each participant completed the questionnaire twice, with a 1-month interval between administrations. The questionnaires were appropriately coded and distributed along with instructions to ensure that the second administration could be matched with the first.

Inclusion criteria consisted of having at least 1 year of professional nursing experience and willingness to participate in both rounds of the survey. The exclusion criterion was the inability to complete the questionnaire during the second stage of validation.

### 2.5 Ethical procedure

The study was conducted in accordance with ethical standards outlined in the Declaration of Helsinki (64th WMA General Assembly, Fortaleza, Brazil, October 2013) and Polish legal regulations. Approval was obtained from the Bioethics Committee of PANS in Przemyśl (approval number: KBPANS/10/2024).

### 2.6 Statistical analysis

To validate the Polish version of the Soft Skills Questionnaire for Nurses (SSQN), its internal structure, reliability, and psychometric properties were thoroughly evaluated. Internal consistency was assessed using Cronbach's alpha coefficient to determine the reliability of each subscale within the questionnaire. Test-retest reliability was examined by comparing two sets of responses using descriptive statistics and the Wilcoxon test to evaluate the significance of any differences. Additionally, Spearman's rank correlation coefficient was applied to assess the strength and direction of associations between the two administrations.

A significance level of *p* < 0.05 was adopted as the criterion for statistical significance when interpreting the test results. All analyses were performed using STATISTICA software, version 13.

To identify measures describing soft skills competencies, an exploratory factor analysis was conducted. Prior to this, the presence of correlations among the 25 detailed items was verified using the Kaiser-Meyer-Olkin (KMO) criterion and Bartlett's test of sphericity. The results were satisfactory, with a KMO value of 0.788 (where a value of 0.7 is generally considered sufficient for factor analysis). A statistically significant Bartlett's test result (*p* < 0.001) allowed rejection of the null hypothesis of no correlations among the detailed items, thus confirming the suitability of the data for factor analysis.

## 3 Results

Of the 600 questionnaires distributed, 523 were returned. Following a completeness check, 496 were deemed valid and included in the final analysis, resulting in an effective response rate of 82.6%. The sample size had been estimated to ensure that, if they obtained Cronbach's alpha reached 0.75, it would significantly exceed the commonly accepted validation threshold of 0.70. With a statistical power of 0.85 and a significance level of 0.05, the minimum required sample size was calculated at 447. Thus, the final number of 496 valid responses not only met but exceeded this criterion, ensuring sufficient statistical robustness.

The study sample consisted of 496 nurses–472 women and 24 men—with diverse ages and levels of professional experience. A detailed demographic and occupational profile of the participants is provided in Table 1.

**Table 1:** Characteristics of the study group.

**Variable**	**Category**	**Frequency (*N*)**	**Percentage (%)**
Gender	Man	24	4.8
	Woman	472	95.2
Age (yrs.)	< 25	25	5.0
	25–35	66	13.3
	36–45	108	21.8
	46–55	177	35.7
	>55	120	24.2
Work experience (yrs.)	< 5	52	10.5
	5–10	72	14.5
	11–20	110	22.2
	>20	262	52.8
Education	High school/medical school	117	23.6
	Bachelor in Nursing	170	34.3
	Master in Nursing	209	42.1
Communicative knowledge of foreign languages	English	209	42.1
	German	38	7.7
	Russian	109	22.0
	Italian	6	1.2
	Lack of language skills	134	27.0
Assessment of communication skills	Acceptable	72	14.5
	Good	214	43.1
	Very good	193	38.9
	Excellent	17	3.4

### 3.1 Soft skills questionnaire

#### 3.1.1 Description of the questionnaire—how to calculate numerical measures

The Soft Skills Questionnaire for Nurses comprises 25 items designed to evaluate nurses' preferred behaviors during interactions with patients and co-workers. Respondents answered each question using a 5-point Likert scale, where 1 indicated “strongly disagree” and 5 indicated “strongly agree.” This scale allowed the quantification of soft skills by assessing levels of agreement with various behavioral statements.

The questionnaire measures three key dimensions of nursing soft skills:

- Nursing Communication Skills (NCS): This dimension evaluates communication abilities using 13 questions (questions 1, 2, 3, 4, 5, 6, 7, 9, 10, 11, 17, 21, and 22). The NCS score is calculated as the sum of responses to these items, with possible values ranging from 13 to 65 points.
- Confidentiality (CON): This dimension assesses confidentiality practices through six questions (questions 15, 18, 19, 20, 24, and 25). The total CON score ranges from 6 to 30 points.
- Management and Emotional Intelligence (MEI): This dimension evaluates the ability to manage responsibilities and demonstrate emotional intelligence using six questions (questions 8, 12, 13, 14, 16, and 23). The MEI score also ranges from 6 to 30 points.

In addition to these individual measures, the Total Nursing Soft Skills (TNSS) score is calculated as the sum of all 25 items, with a possible range of 25–125 points. This comprehensive measure reflects the overall level of a nurse's soft skills across all dimensions. When calculating TNSS values, it is important to account for certain statements that reflect inappropriate nursing attitudes. For example, statements such as “I come to patients' rooms only when the patient requests it (8)” or “It is not necessary to ask for the patient's consent before starting a new treatment (20)” require careful handling. These specific items were highlighted in the questionnaire's description table (Appendix A), and their scoring scales were inverted to align with the desired positive attitudes before calculating the NCS, CON, and MEI measures.

Appendix A provides a detailed list of all 25 questionnaire items, their corresponding measures (NCS, CON, or MEI), and notes on items requiring scale inversion. This ensures clarity and consistency in scoring and interpretation.

#### 3.1.2 Results of soft skills survey

The table below presents the characteristics of the assessment of the three components of soft skills, as well as the summary measure for the study population. Interpreting these results is challenging, as establishing a reference point will require additional published research. However, these values may serve as a guide for future studies using the same questionnaire to assess nurses' soft skills in other regions or countries.

Despite the difficulty in assigning absolute values, a preliminary interpretation of the results is possible. The average score for Nursing Communication Skills (NCS) is 52.0 points, which represents 75% of the maximum possible score. For Confidentiality (CON), the average is 19.4 points, corresponding to 56% of the maximum score, while the Management and Emotional Intelligence (MEI) measure averages 20.2 points, equaling 59% of the maximum possible score. Finally, the Total Nursing Soft Skills (TNSS) summary score averages 91.7 points, which is 67% of the maximum score.

In summary, the communication skills scores are above average, while the scores for confidentiality and management/emotional intelligence are closer to average (Table 2).

**Table 2:** Questionnaire results.

**Soft skills measures**	**Mean**	**Median**	**Std. dev**.	**Min**	**Max**	**Skewness**
Nursing communication skills	52.0	52.0	4.9	38	65	−0.12
Confidentiality	19.4	20.0	4.3	6	30	−0.56
Management and emotional intelligence	20.2	21.0	3.5	9	30	−0.43
Total nursing soft skills	91.7	92.0	8.1	71	112	−0.14

It is important to note that the creators of the original questionnaire did not provide specific values for the individual measures (NCS, CON, MEI) or the summary measure (TNSS), focusing instead on the validation of the tool itself. This publication is the first to detail the method for calculating these measures and to present specific results, derived from a large sample.

#### 3.1.3 Validation of the SSQN questionnaire

##### 3.1.3.1 Internal consistency

The internal consistency of the NCS, CON, and MEI measures was assessed using Cronbach's alpha statistic.^1^ Tables 2–4 present the mean and standard deviation for the responses to each individual statement within the measures, along with the Cronbach's alpha value representing the internal consistency of the summary measure. Additionally, for each statement, the Cronbach's alpha value recalculated after excluding that statement from the questionnaire is provided. If removing a statement significantly increases the alpha value, it suggests that the statement's exclusion from the questionnaire should be considered.

The internal consistency of the NCS questionnaire is relatively high (α = 0.669), though slightly below the commonly accepted threshold of 0.70 for satisfactory reliability. However, given the small difference, the validation outcome can be considered acceptable. Excluding any individual statement has minimal impact on the overall alpha value, which remains within a narrow range (0.635–0.682).

The acceptance level for individual statements within the NCS is generally high, with the highest score observed for Q3 (4.47) and the lowest for Q9 (2.86), as shown in Table 3.

**Table 3:** Descriptive statistics for the acceptance of statements included in the NCS measure, along with an assessment of the internal consistency of the summary measure using Cronbach's alpha.

**Item**	**Mean**	**Standard deviation**	**Cronbach's alpha**
Q1	4.52	0.62	0.635
Q2	3.90	0.92	0.646
Q3	4.63	0.59	0.643
Q4	4.47	0.59	0.635
Q5	3.66	1.05	0.670
Q6	3.59	1.05	0.654
Q7	4.17	0.72	0.639
Q9	2.86	1.12	0.682
Q10	4.09	0.82	0.644
Q11	4.35	0.59	0.637
Q17	3.91	0.94	0.663
Q21	3.94	0.92	0.660
Q22	3.94	0.84	0.650
Summary measure	52.02	4.94	0.669

The internal consistency of the questions included in the confidentiality assessment (CON) is lower compared to the NCS, with a Cronbach's alpha value of 0.601. Analyzing the Cronbach's alpha values after removing individual items reveals that excluding question 24 would slightly improve the overall consistency. However, the improvement is modest, raising the α value to 0.643 if this item were excluded (Table 4).

**Table 4:** Descriptive statistics for acceptance of statements included in the CON measure, along with an assessment of the consistency of the internal summary measure using Cronbach's alpha.

**Item**	**Mean**	**Standard deviation**	**Cronbach Alpha**
Q15	3.19	1.19	0.594
Q18	2.71	1.41	0.529
Q19	2.53	1.29	0.510
Q20	1.92	1.12	0.525
Q24	3.78	1.16	0.643
Q25	2.43	1.27	0.510
Summary measure^*^	19.42	4.31	0.601

^*^Since the CON summary measure was calculated after inverting the scale for some of the component questions, the mean for the summary measure is not the sum of the component means.

The management and emotional intelligence (MEI) section of the questionnaire exhibited the lowest internal consistency, with a Cronbach's alpha value of just 0.544. Detailed analysis suggests that removing question 23 would improve internal consistency, increasing the α value to 0.611 (Table 5).

**Table 5:** Descriptive statistics for acceptance of the statements included in the MEI measure, along with an assessment of the consistency of the internal summary measure using Cronbach's alpha.

**Item**	**Mean**	**Standard deviation**	**Cronbach alpha**
Q8	2.17	1.08	0.455
Q12	2.66	1.08	0.443
Q13	3.83	0.92	0.555
Q14	2.44	1.06	0.424
Q16	2.54	1.25	0.443
Q23	3.88	0.88	0.611
Summary measure^*^	20.24	3.49	0.544

^*^Since the CON summary measure was calculated after inverting the scale for some of the component questions, the mean for the summary measure is not the sum of the component means.

The Cronbach's alpha value for the entire questionnaire (i.e., the TNSS measure) was 0.644.

##### 3.1.3.2 Repeatability

An essential aspect of validating any psychometric measure is assessing its repeatability. Measurements taken from the same group of participants after a reasonable time interval should produce similar results. Naturally, these results will not be identical, as factors such as the respondents' mood at the time of testing can influence their answers. However, the differences between the two measurement series should remain relatively small and free from systematic patterns.

To evaluate the repeatability of the assessed questionnaire, a follow-up survey was conducted with the same group of participants after 1 month.

The consistency between the two measurement series for the NCS, CON, MEI, and TNSS measures was assessed by comparing descriptive statistics for both rounds of measurements and their differences. The significance of the differences was analyzed using the Wilcoxon test, while Spearman's rank correlation coefficient was used to evaluate correlation.

Additionally, the Intraclass Correlation Coefficient (ICC) was used to assess consistency, with ICC values ranging from 0.0 to 1.0. ICC values between 0.6 and 0.8 indicate good repeatability, while values above 0.8 are considered excellent.

The results of the analysis were consistently positive across all statistical tools. The Wilcoxon test indicated no systematic differences between the test and retest (*p* > 0.05). Spearman's correlation coefficient showed a very high correlation between the two measurement series (*r*_*s*_ > 0.90), and the ICC was also very high (ICC > 0.90).

Thus, it can be confidently concluded that the nursing soft skills questionnaire demonstrates strong repeatability and stability of results (Table 6).

**Table 6:** Repeatability of results.

**Variable**	**Measurement**	**Mean**	**Median**	***s***	**min**	**max**	***p* ^a^**	** $r_{\text{S}}^{\text{b}}$ **	**ICC^c^**
Nursing communication skills	Test	52.02	52	4.94	38	65	0.6866	0.93	0.9193
	Re-test	51.99	52	4.99	35	65			
	Re-test vs. test	−0.02	0	2.00	−14	12			
Confidentiality	Test	19.42	20	4.31	6	30	0.1605	0.93	0.9328
	Re-test	19.37	20	4.31	6	30			
	Re-test vs. test	−0.07	0	1.58	−10	16			
Management and emotional intelligence	Test	20.24	21	3.49	9	30	0.4017	0.94	0.9329
	Re-test	20.20	21	3.52	9	30			
	Re-test vs. test	−0.05	0	1.29	−10	12			
Total nursing soft skills	Test	91.69	92	8.14	71	112	0.2486	0.94	0.9347
	Re-test	91.56	92	8.36	69	114			
	Re-test vs. test	−0.14	0	2.99	−20	28			

^a^p-value calculated using Wilcoxon test.

^b^Spearman's correlation coefficient.

^c^ICC—Intraclass Correlation Coefficient.

An additional part of the repeatability analysis is a comparison of the number and percentage of individuals for whom identical results were obtained in both tests. The high percentage (around 70%) of consistent results is important, but equally notable is the similar proportion of smaller and larger results in the re-test (Table 7).

**Table 7:** Re-test.

**Re-test vs. test**	**Nursing communication skills**		**Confidentiality**		**Management and emotional intelligence**		**Total nursing soft skills**	
	***N***	**%**	***N***	**%**	***N***	**%**	***N***	**%**
Decrease	73	15.0	58	11.9	50	10.3	91	18.7
No change	346	71.2	390	80.2	390	80.2	318	65.4
Increase	67	13.8	38	7.8	46	9.5	77	15.8

**Figure 1:**
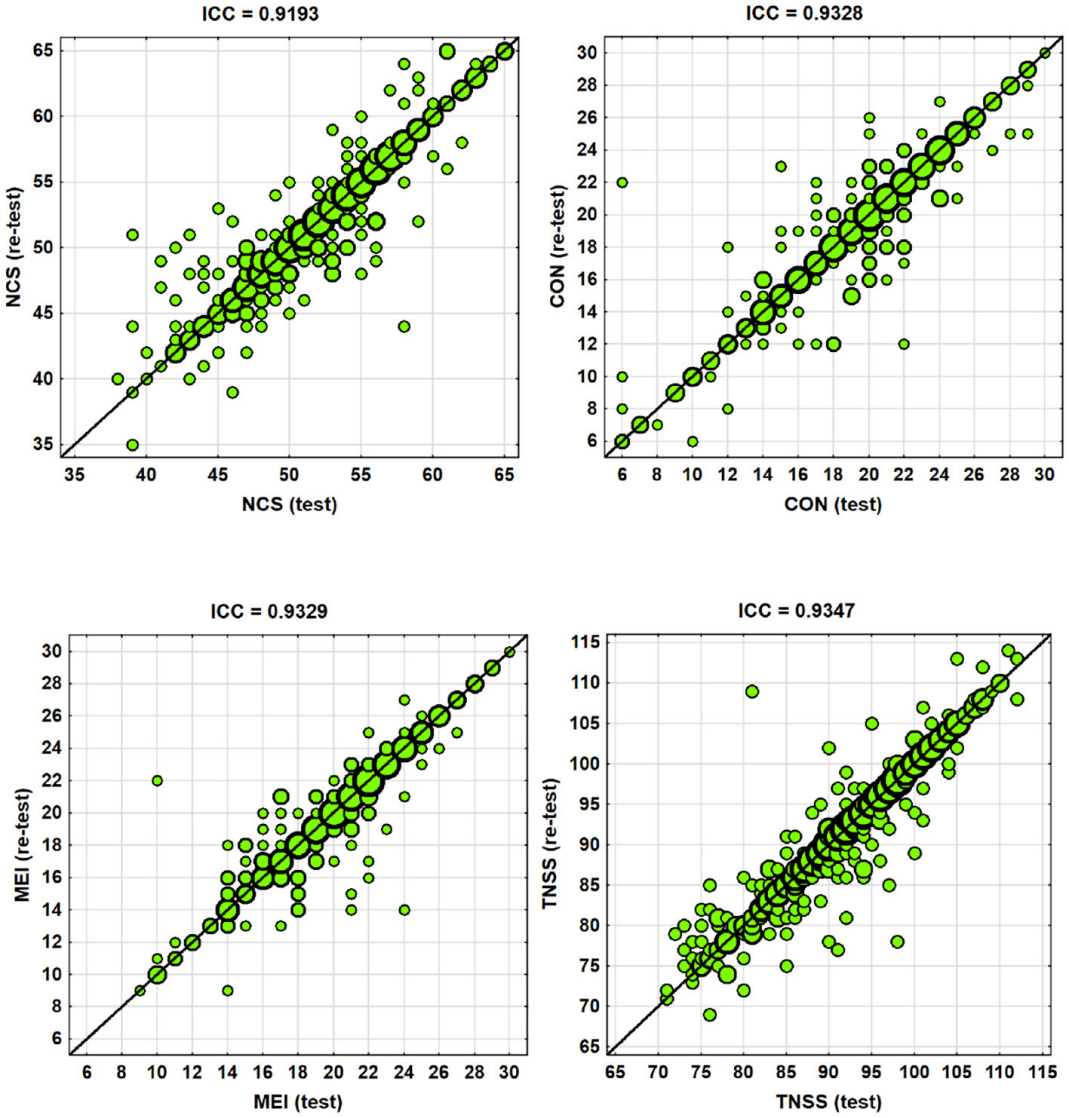
Visualizations of the test vs. re-test study.

The visualizations of the test vs. retest study are presented in scatter plots, where a high repeatability of results for both measurement series is clearly visible for the majority of respondents, with relatively small differences that are randomly distributed—both positive and negative (Figure 1).

A total of 496 nurses participated in the SSQN validation study. The reliability analysis demonstrated moderate internal consistency for the subscales: NCS (communication): α = 0.669, CON (confidentiality): α = 0.601, MEI (management and emotional intelligence): α = 0.544. The entire questionnaire (TNSS) showed an overall Cronbach's alpha of α = 0.644.

Although these alpha values fall below the optimal threshold of 0.70, the instrument demonstrated excellent measurement stability (ICC > 0.90) based on a 1-month test–retest procedure, indicating strong temporal reliability. No significant differences were found between the first and second measurements (Wilcoxon test, *p* > 0.05), and a high Spearman correlation (*r*_*s*_ > 0.90) was observed between the measurement series.

Based on these findings, the SSQN can be considered a reliable and stable tool, suitable for further analyses and adaptation.

## 4 Discussion

The aim of this paper was to present the process of adapting the psychometric properties of the Polish version of the “Soft Skills Questionnaire for Nurses”. The translated Nursing Soft Skills Questionnaire, like its original version, consists of 25 questions in which nurses were asked about their preferred behaviors during different forms of patient care and interactions with colleagues. Responses to each question were given using a 5-point Likert scale, which determined the level of acceptance of a particular behavior toward patients and colleagues (1—strongly disagree, …, 5—strongly agree). The authors of the questionnaire identified three groups of questions based on which the assessment of communication skills (NCS—Nursing Communication Skills), confidentiality (CON—Confidentiality), and management and emotional intelligence (MEI—Management and Emotional Intelligence) were determined.

The assessment of communication skills, confidentiality, and management and emotional intelligence was calculated as the sum of points for the individual questions. Due to the different number of questions in each group, the score range for the NCS measure was from 13 to 65 points, while for C and MEI it ranged from 6 to 30 points. Based on the responses to all 25 questions, a total soft skills score (TNSS—Total Nursing Soft Skills) can also be calculated, with values ranging from 25 to 125 points.

When calculating the TNSS value, it is important to note that some statements refer to inappropriate attitudes of nurses [for example, “I only go to patient rooms when the patient requests it (8)” or “It is not necessary to ask for the patient's consent before starting new treatment (20)”]. These questions were highlighted in a table containing a description of the questionnaire (Appendix A), and before calculating the NCS, C, and MEI measures, their scale was reversed.

Based on the analysis results, it can be confidently stated that the evaluated nursing soft skills questionnaire demonstrates repeatability, stability of results, and can be used for scientific research.

There are only a few publications in the literature that address the issue of soft skills within the nursing profession. Based on interviews with Indonesian nurses, Ernawati (20) identified nine core interpersonal skills essential for novice nurses. The results of their study provide a foundation for the development of soft skills in this professional group. They also recommended the inclusion of soft skills training in nursing education programs.

Similarly, findings by Laari et al. (22) highlight an urgent need for both the development and assessment of soft skills in the field of nursing. The study conducted by Atalla et al. (23) demonstrated that nurses highly valued soft skills and were aware that interpersonal abilities can enhance patient care, increase job satisfaction, and contribute to overall organizational success.

In addition to care recipients—namely patients and their family members—Ng (24) emphasized that soft (service-oriented) skills are crucial not only for the success of healthcare organizations but also for the effectiveness of healthcare professionals themselves.

In the article, the authors presented the overall Cronbach's alpha values for each scale; however, a detailed identification of items with the lowest internal consistency is also warranted. In the NCS subscale (communication), item Q9 (“If the patient does not want to talk, I do not insist”) had the lowest mean response value, which may indicate cultural ambiguity or variability in interpretation. The alpha coefficient increased to 0.682 upon removing this item—the highest among all analyzed variants—suggesting that Q9 has the strongest negative impact on the coherence of this subscale.

In the CON subscale (confidentiality), the most notable increase in alpha following item removal was observed for Q24 (“I store documentation in areas accessible only to authorized personnel”), with the coefficient rising to 0.643. Although the difference is not substantial, it may indicate that Q24 is semantically inconsistent with the rest of the subscale or measures a different dimension of behavior (e.g., organizational rather than personal).

Within the MEI subscale (management and emotional intelligence), item Q23 (“I always control my emotions in difficult situations”) had the greatest negative effect on internal consistency. Its removal raised the alpha from 0.544 to 0.611, suggesting that Q23 may be perceived as overly general or normative, leading to less varied or less candid responses.

In light of these findings, the authors plan to conduct further content analysis of these items in subsequent stages of tool development. This will involve rephrasing or replacing ambiguous items with clearer and more differentiated ones. They are also considering expanding the subscales, which may further improve reliability, particularly for the MEI and CON dimensions.

The validation results indicate that the SSQN has the potential to be a useful instrument for assessing nurses' soft skills. Nevertheless, further development should address several key directions, such as cross-validation and criterion-related validation. It will be essential to test the model on an independent sample to confirm the stability of the factor structure and the overall validity of the instrument. Cross-validation using, for example, CFA on a different group will help assess the replicability of the theoretical assumptions.

Future studies will explore the relationship between SSQN scores and external indicators of nursing performance (e.g., supervisor evaluations, patient satisfaction, frequency of interpersonal conflicts). This will help determine whether higher SSQN scores are indeed associated with desirable professional outcomes. Expanding the subscales with additional items may be necessary to improve reliability—especially for MEI and CON. Simultaneously, it would be valuable to consider the use of structural equation modeling (SEM) to better understand the relationships between soft skills and other occupational or demographic variables.

A promising direction also includes the adaptation and validation of the SSQN in other countries and cultural contexts, which may contribute to the development of a cross-culturally comparable tool.

## 5 Conclusion

The questionnaire distinguished three groups of questions, based on which the assessment of communication skills (NCS—Nursing Communication Skills), confidentiality (CON—Confidentiality), and management and emotional intelligence (MEI—Management and Emotional Intelligence) were determined. The internal consistency of the NCS-related questions is fairly high (0.669), although slightly below the 0.70 threshold, which is considered acceptable. The internal consistency of the questions related to confidentiality (CON) is lower than for NCS, with a Cronbach's alpha value of 0.601. The lowest internal consistency is found in the section of the questionnaire on management and emotional intelligence (MEI), with a Cronbach's alpha value of only 0.544. In the analysis, all statistical tools yielded positive results—the Wilcoxon test did not show systematic differences between the test and retest (*p* > 0.05), the Spearman correlation between the two measurement series was very high (*r*_S_ > 0.90), and the ICC was also very high (ICC > 0.90). The assessed nursing soft skills questionnaire demonstrates repeatability, stability of results, and can be used for scientific research.

## 6 Limitations of the study

The results of the internal consistency analysis are not fully satisfactory, as the Cronbach's alpha values for the NCS, CON, and MEI measures were below the commonly accepted threshold of 0.70. For the NCS measure, the difference was not significant (Cronbach's alpha of 0.669). The lowest internal consistency was observed in the questionnaire section on management and emotional intelligence (MEI), with a Cronbach's alpha of 0.544. However, the authors ultimately recommend considering these results as positive, because the methodology for determining Cronbach's alpha tends to produce lower values for measures based on a small number of questions, such as CON and MEI, which are determined based on responses to 6 constituent questions. The sample includes only two hospitals, based on the assumption that their functional diversity and the wide range of represented positions and professional experience would allow for the collection of data with generalizable characteristics. Nevertheless, the authors acknowledge the potential limitations in representativeness due to environmental factors or local organizational culture.

## 7 Implications for practice

The use of standardized questionnaires to assess soft skills among nurses is both justified and necessary. The current lack of validated tools, combined with reliance on literature reviews and interviews conducted in small samples, limits the ability to accurately assess the current level of interpersonal competencies. Findings derived from standardized instruments may serve as a foundation for introducing changes in the educational standards for future nurses. Such modifications would enable academic educators to more effectively develop soft skills among students, thereby contributing to their comprehensive preparation for professional practice.

## Data Availability

The raw data supporting the conclusions of this article will be made available by the authors, without undue reservation.

## Footnotes

^1^The Cronbach's alpha statistic is a measure of the internal consistency (internal consistency) of the questions comprising the questionnaire. The value of this statistic can range from 0 (absolute lack of consistency in the answers to the component questions) to 1 (perfect consistency). In many publications, a scale is assumed to be internally consistent when this measure is no < 0.70.

## Appendix

**Table A1:** Questions included in the soft skills questionnaire for nurses.

**No**	**Statement**	**Degree of acceptance of the statement** ^******^				
1	I usually greet my patients when checking on them	1	2	3	4	5
2	I always introduce myself to my new patients	1	2	3	4	5
3	Once forgetting my patient's name, I either use Mr. or Mrs. to identify the patient	1	2	3	4	5
4	I rarely forget to introduce my role as a nurse to the patient	1	2	3	4	5
5	Written technique can't be used to answer patient's inquiries	1	2	3	4	5
6	I never interrupt my peers during their lunch breaks	1	2	3	4	5
7	I demonstrate steps and actions to help my patient understand any new procedure	1	2	3	4	5
8^*^	I only show up in the patient room, upon my patient's call	1	2	3	4	5
9	I usually prefer to discuss the patient's critical case with his/her family, rather than with the patient him/herself	1	2	3	4	5
10	It is important to have the patient express his/her case before formulating a care plan	1	2	3	4	5
11	I always establish active listening while in the patient's room	1	2	3	4	5
12^*^	I do not prefer to interfere when a peer is facing a problem (worries, needs, personal concerns)	1	2	3	4	5
13^*^	I am sometimes aware of my basic needs (hunger, thirst…) to ensure excellent patient service	1	2	3	4	5
14^*^	I am not attentive to a patient's non-justifying behavior (uncontrolled emotions), when am suffering from time shortage	1	2	3	4	5
15^*^	It is preferable to initiate one-to-one conversation with the patient about his/her personal life.	1	2	3	4	5
16^*^	Presenting the necessary guidance to the patient is not the nurse's responsibility	1	2	3	4	5
17	I always smile when delivering any type of care or even news to my patient	1	2	3	4	5
18^*^	The patient's privacy is not necessary in all situations	1	2	3	4	5
19^*^	I always separate men and women in patients' room just for medical purposes	1	2	3	4	5
20^*^	It is not necessary to ask for the patient's permission, when starting any new treatment	1	2	3	4	5
21	I try not to raise my voice when calling my peers	1	2	3	4	5
22	I try not to ignore the question when not updated with the patient's case	1	2	3	4	5
23	I always ask the patient if the physician visited and checked on him/her	1	2	3	4	5
24^*^	I don't address the first degree family members with the patient's medical status	1	2	3	4	5
25^*^	I don't believe that mutual trust should be built with the patient	1	2	3	4	5

^*^Statements of a negative nature require the scale direction to be reversed to calculate the summary measure.

^**^1—strongly disagree, 2—disagree, 3—neutral, 4—agree, 5—strongly agree.
